# Extracts Prepared from a Canadian Toxic Plant Induce Light-Dependent Perinuclear Vacuoles in Human Cells

**DOI:** 10.3390/toxins13020138

**Published:** 2021-02-12

**Authors:** Jan M. Tuescher, Chad R. Beck, Locke Spencer, Benjamin Yeremy, Yutong Shi, Raymond J. Andersen, Roy M. Golsteyn

**Affiliations:** 1 Natural Product and Cancer Cell Laboratories, University of Lethbridge, Lethbridge, AB T1K 3M4, Canada; j.tuescher@uleth.ca (J.M.T); chad.beck@uleth.ca (C.R.B); 2 Department of Physics and Astronomy, University of Lethbridge, Lethbridge AB T1K 3M4, Canada; locke.spencer@uleth.ca; 3 Department of Earth, Ocean, Atmospheric Sciences, University of British Columbia, Vancouver, BC V6T 1Z4, Canada; yeremy@chem.ubc.ca (B.Y.); sytmarine@zju.edu.cn (Y.S.); raymond.andersen@ubc.ca (R.J.A.)

**Keywords:** Canada, Caprifoliaceae, laminopathies, lamins A/C, nuclear associated vacuole (NAV), nucleus, Pelger-Huët Anomaly, *Symphoricarpos occidentalis*

## Abstract

**Abstract:**

We are investigating plant species from the Canadian prairie ecological zone by phenotypic cell assays to discover toxins of biological interest. We provide the first report of the effects of extracts prepared from the shrub *Symphoricarpos occidentalis* in several human cell lines. *S. occidentalis* (Caprifoliaceae) extracts are cytotoxic, and, strikingly, treated cells undergo light-dependent vacuolation near the nucleus. The range of irradiation is present in standard ambient light and lies in the visible range (400-700 nm). Vacuolization in treated cells can be induced with specific wavelengths of 408 or 660 nm at 1 J/cm^2^ energies. Vacuolated cells show a striking phenotype of a large perinuclear vacuole (nuclear associated vacuole, NAV) that is distinct from vesicles observed by treatment with an autophagy-inducing agent. Treatment with *S. occidentalis* extracts and light induces an intense lamin A/C signal at the junction of a nuclear vacuole and the nucleus. Further study of *S. occidentalis* extracts and vacuolation provide chemical tools that may contribute to the understanding of nuclear envelope organization and human cell biology.

**Key Contribution:**

We provide the first description of the biological effects upon human cells of extracts from the toxic plant, *Symphoricarpos occidentalis*. Treated cells acquire striking nuclear associated vacuoles (NAVs), rarely observed in animal cell biology.

## 1. Introduction

Canada harbours 15 terrestrial ecozones including the prairie zone, which has one of the highest levels of biodiversity in temperate northern latitudes [1]. Despite extensive research into plant natural products worldwide, the prairie ecological zones are rarely investigated for plants with bioactive secondary metabolites [2,3,4]. Prairie plant species have coevolved with both abiotic and biotic stresses, such as the prevalence of snowpacks that shorten the growing season, and the presence of grazing herbivores [5]. These factors increase competition for survival between plant species, which contributes to the production of secondary metabolites [6]. Furthermore, prairie plants have been used by Indigenous peoples of Canada as a source of health materials [2]. In one study of a library of 35 Canadian medicinal plants, extracts were tested for anticancer activities, of which 11 were potent inducers of cell death [4]. These observations and the abundance of untested plant species suggests that investigating prairie plant species could lead to the discovery of compounds with novel biological activities. Frequently, these chemicals can become unique tools with which to investigate cellular pathways and increase our knowledge about connections in biology.

There are two major approaches to investigate the biological activity of plant extracts or chemicals. One approach, known as the targeted approach, is directed to identifying chemicals that modulate specific enzymes in biological pathways. For example, in cancer research, chemicals from both synthetic and natural product sources have been screened widely against enzymes such as protein kinases. A second approach, known as phenotypic assays, applies chemicals and extracts to cells and then observes them for a response. This approach is more likely to lead to novel observations because it is not biased, relative to the targeted approach. Examples of this approach have been particularly successful in analysis of natural products from marine organisms [7]. Phenotypic and targeted approaches complement one another, and eventually the outcome of a phenotypic approach may lead to a research program directed to a newly discovered target [8].

**Figure 1:**
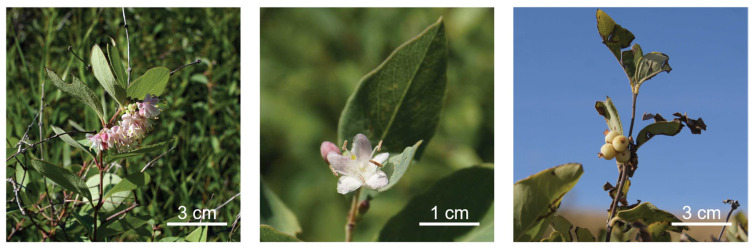
*Symphoricarpos occidentalis* (Caprifoliaceae) in prairie habitat (**left**). Flower and leaf detail (**middle**); Fruits (**right**).

We are investigating plants from the prairie ecological zone to discover new cell biology tools with which to study how human cells function. We have previously reported that extracts prepared from several prairie plant species, including *Thermopsis rhombifolia* that induced a cell cycle arrest identified by phenotypic assays [3,9]. Here, we identified extracts prepared from *Symphoricarpos occidentalis* (Caprifoliaceae) that induce a striking cellular phenotype when applied to human cells. *Symphoricarpos occidentalis*, commonly known as the Snowberry or Wolfberry, is native to the prairie ecological zone in Canada [10,11] (Figure 1). It grows as dense shrubs to a height of 1 metre with abundant leaves of 3–5 cm in length. The common name Snowberry refers to the pale fruit produced by autumn. Cells treated with *S. occidentalis* extracts acquire a photo-inducible, perinuclear vacuole (also known as a nuclear associated vacuole, NAV). The formation of perinuclear vacuoles is unusual in animal cell biology, and we distinguished these from vesicles produced by autophagy. By the phenotypic assay approach of a previously unstudied prairie plant species, we identified an extract that may become a tool for future cell biology studies of toxicity and nucleus structure.

## 2. Results

**Figure 2:**
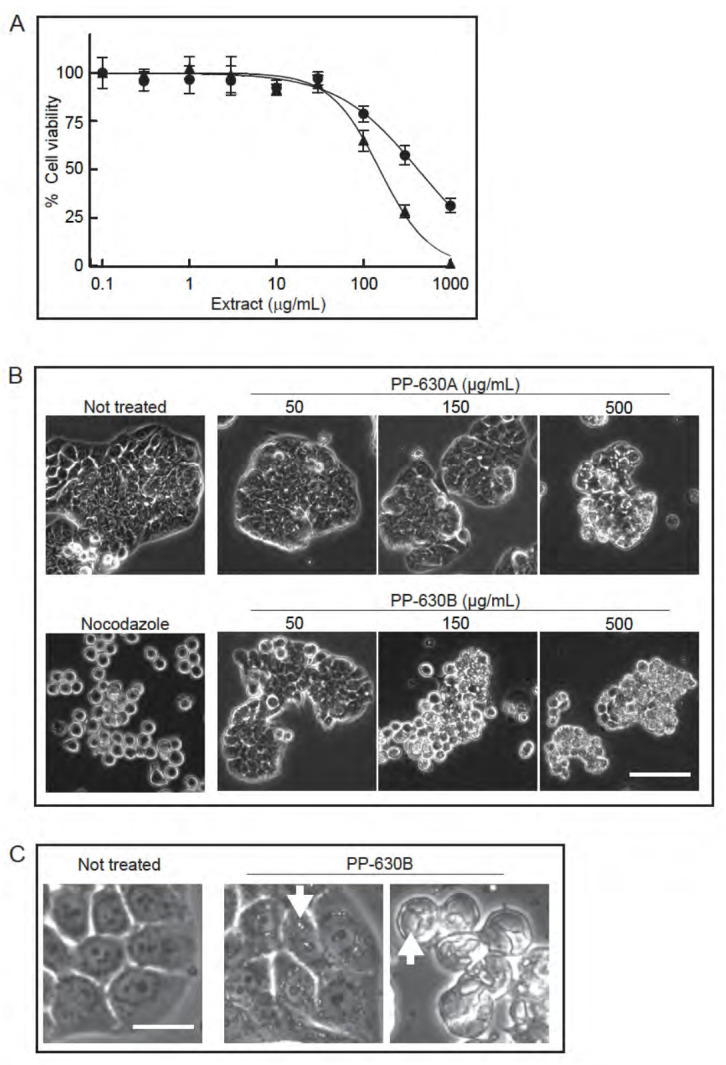
Extracts prepared from *S. occidentalis* leaves are toxic to HT-29 cells. (**A**) HT-29 cells were treated with increasing concentrations of either PP-630A (circles) or PP-630B (triangles) for 96 h and cell viability was measured by the MTT assay. Mean percentages of viability were calculated and standard errors of the means are shown. The mean IC50 concentrations of PP-630A were 426 ± 81 µg/mL and of PP-630B were 154 ± 34 µg/mL. (**B**) HT29 cells were not treated, treated with nocodazole, or treated with a range of concentrations of PP-630A or PP-630B. Representative images taken by phase contrast microscopy at 24 h are shown. Scale bar = 50 μm. (**C**) HT29 cells were either not treated or treated with 150 μg/mL PP-630B for 24 h and observed by phase contrast microscopy to show cell detail. Representative images of vacuole like structures (arrows) are shown. Scale bar = 10 μm.

We extracted *S. occidentalis* leaves (Figure 1) with either 75% ethanol/water (*v*/*v*) (PP-630A) or with 100% dichloromethane (PP-630B) and tested if the extracts were toxic to HT-29 cells using the MTT assay (Figure 2A). The IC50 for PP-630A was 426 ± 81 μg/mL and for PP-630B was 154 ± 34 μg/mL. We observed the treated HT-29 cells at 24 h by phase-contrast microscopy to determine if the cell morphology had changed prior to cell death. HT-29 cells were not treated or treated with nocodazole, a compound that induces a rounded phenotype by arresting cells in mitosis [12]. We also treated cells with a range of concentrations of PP-630A or PP-630B. The not-treated sample displayed the standard morphology of a few rounded cells and clusters of cells typical of a polarized epithelial cell line, whereas nocodazole-treated cells showed a round morphology, as expected (Figure 2B). PP-630A-treated cells showed a normal morphology at 50 and 150 µg/mL and an aberrant morphology with vacuole-like structures in cells at 500 µg/mL. PP-630B-treated cells showed little change at 50 µg/mL, whereas treatment at 150 and 500 µg/mL showed aberrant morphologies including the presence of vacuole-like structures (Figure 2B,C). These results revealed that extracts prepared from *S. occidentalis* leaves are cytotoxic and induce aberrant morphologies when applied to HT-29 cells.

**Figure 3:**
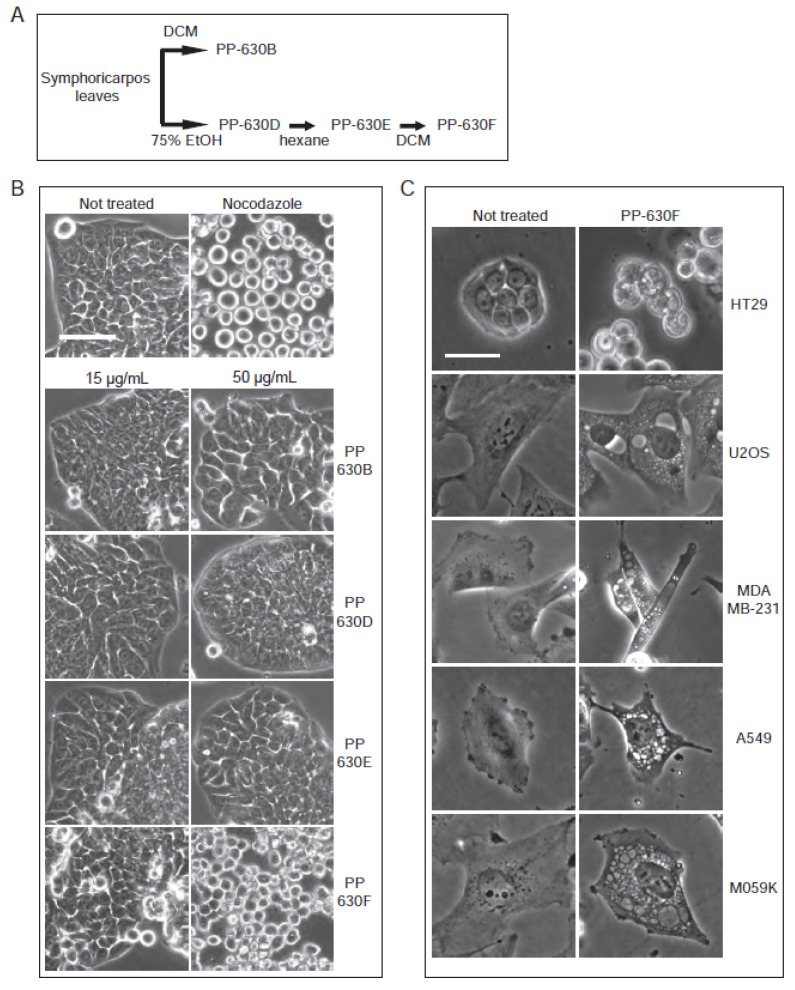
Extracts of *S. occidentalis* leaves produce striking vacuoles in a variety of treated cell lines. (**A**) Scheme of the extraction of *S. occidentalis* leaves with different solvents and in a sequence. (**B**) HT-29 cells were not treated, treated with nocodazole, or treated with 15 or 50 µg/mL of PP-630B, PP-630D, PP-630E, or PP-630F (seq. DCM) for 24 h and observed by phase-contrast light microscopy. Scale bar = 50 µm. (**C**) Different cell lines were either not treated (left) or treated with 50 µg/mL of PP-630F (right) and imaged at 24 h by phase contrast microscopy. Scale bar = 50 µm.

Extract PP-630B, which was more toxic than extract PP-630A, was oily and therefore difficult to weigh and solubilize reliably. We then prepared a sequential extraction of *S. occidentalis* leaves with 75% (*v*/*v*) ethanol/water (PP-360D) followed by 100% hexane (PP-630E) before a final extraction with 100% dichloromethane, giving rise to a non-oily extract PP-630F (Figure 3A). We then tested if PP-630F was still cytotoxic and induced the clear zone structures observed by PP-630A treatment. HT-29 cells were not treated, treated with nocodazole, or treated with 15 or 50 µg/mL of PP-630B, PP-630D, PP-630E, or PP-630F and observed by phase contrast microscopy. Nocodazole-treated cells showed a round phenotype (Figure 3B), whereas PP-630F-treated cells showed aberrant morphology at 50 µg/mL compared to extracts PP-630B, PP-630D, or PP-630E. Removal of ethanolic and hexane soluble chemicals did not diminish PP-630F capacity to induce clear zones in cells.

We then tested whether treatment with PP-630F induced clear zones in cell lines in addition to HT-29. Cells were not treated or treated with 50 µg/mL of PP-630F (Figure 3B). In addition to HT-29 cells, U2OS (bone osteosarcoma), MDA-MB-231 (breast adenocarcinoma), A549 (lung carcinoma), and M059K (brain glioblastoma) cells were treated in parallel and observed by phase contrast microscopy at 24 h (Figure 3C). Clear zones resembling vacuoles were strikingly visible in each cell line and were easier to observe than in HT-29, in part because of their non-polarized morphology. We selected the U2OS cell line and extract PP-630F for further experiments to investigate the vacuole-like structures.

**Figure 4:**
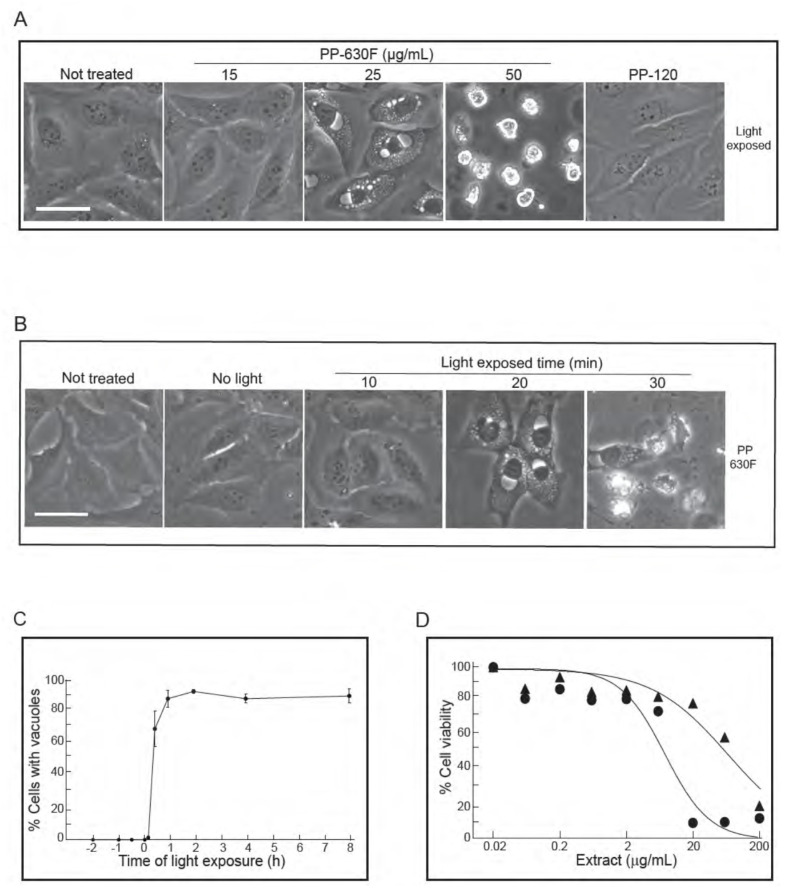
Vacuolation of cells is PP-630F concentration dependent and requires exposure to light. (**A**) U2OS cells were either not treated or treated with 15, 25 or 50 µg/mL of PP-630F and exposed to ambient light. In parallel, cells were treated under similar conditions with extract PP-120 (*Thermopsis rhombifolia*). Cells were imaged at 16 h by phase contrast microscopy. Scale bar = 50 µm. (**B**) U2OS cells were either not treated or treated with 25 µg/mL of PP-630F. Cells were then either kept in the dark (No light) or exposed to ambient light for 10, 20, or 30 min. Cells were imaged at 16 h by phase contrast microscopy. Scale bar = 50 µm. (**C**) U2OS cells were treated with PP-630F at Time 0. Cells were also exposed to ambient light at indicated times before or after extract treatment. Cells were imaged at 16 h after light exposure by phase contrast microscopy. The percentage of cells that had vacuoles in a treated sample were calculated from three experiments and standard errors of the means are shown. (**D**) U2OS cells were treated with increasing concentrations of PP-630F for 96 h and either exposed to ambient light (circles) or kept in the dark (triangles). The MTT assay was used to measure cell viability. Mean percentages of viability were calculated from three experiments and standard errors of the means are shown. The mean IC50 concentration of PP-630F was 8 ± 2 µg/mL and 26 ± 3 µg/mL with and without light, respectively.

During the course of the experiments, we noticed that the induction of the vacuolated phenotype appeared to be linked to the act of handling cells during treatment with PP-630F. Of the methods in handling cultured cells, we eliminated the parameters such as changes in culture temperature, mechanical shock, or pH and discovered that light was required for the vacuolated phenotype. We confirmed this phenomenon by controlling ambient light exposure (Figure 4A). U2OS cells were either not treated or treated with a range of concentrations of PP-630F, then exposed to ambient light and observed by phase-contrast microscopy. In parallel, U2OS cells were treated with extract PP-120, prepared in a similar manner as that of PP-630F from a previously characterized prairie plant species, *Thermopsis rhombifolia* [3] (Figure 4A). Not-treated cells and PP-120-treated cells did not acquire vacuoles, as expected, whereas cells treated with PP-630F had light-induced vacuoles in a dose dependent manner. These data revealed that a chemical(s) in PP-630F was required for vacuole induction.

To test for the requirement for light, U2OS cells were either not treated or treated with 25 µg/mL of PP-630F and either not exposed to ambient light or exposed for different times and observed at 16 h (Figure 4B). In the absence of light or with an exposure of ambient light for 10 min, PP-630F-treated cells did not acquire vacuoles. By contrast, PP-630F treatment and light exposure of 20 min or more induced vacuoles. These data revealed that PP-630F and light were required for vacuole induction.

We then tested the sequence of light and PP-630F treatment by exposing cells to ambient light for 20 min at times ranging from 2 h before PP-630F treatment to 8 h after PP-630F treatment (Figure 4C). The percent of cells with vacuoles were counted at 16 h after light treatment. No cells with vacuoles were observed when cells were exposed to light prior to PP-630F treatment. By contrast, after 30 min of PP-630F treatment, exposure to light induced vacuoles in more than 60% of cells. Finally, in a separate experiment we “primed” cell culture media by co-treatment of cell media with PP-630F and light exposure in the absence of cells. We then added the “primed” media to cells and observed them 16 h later. The “primed” media did not induce vacuoles, demonstrating that light, PP-630F, and the cellular target needed to be present at the same time for the vacuole effect to take place (not shown).

We then measured the viability of U2OS cells treated with PP-630F either without light or with 20 min light exposure by the MTT assay (Figure 4D). The IC50 value of PP-630F against U2OS cells was 65 ± 12 µg/mL in the absence of light and 8 ± 0.5 µg/mL in the presence of ambient light. These results revealed that the combination of PP-630F and light was more cytotoxic than PP-630F alone, likely due to the induced vacuoles.

**Figure 5:**
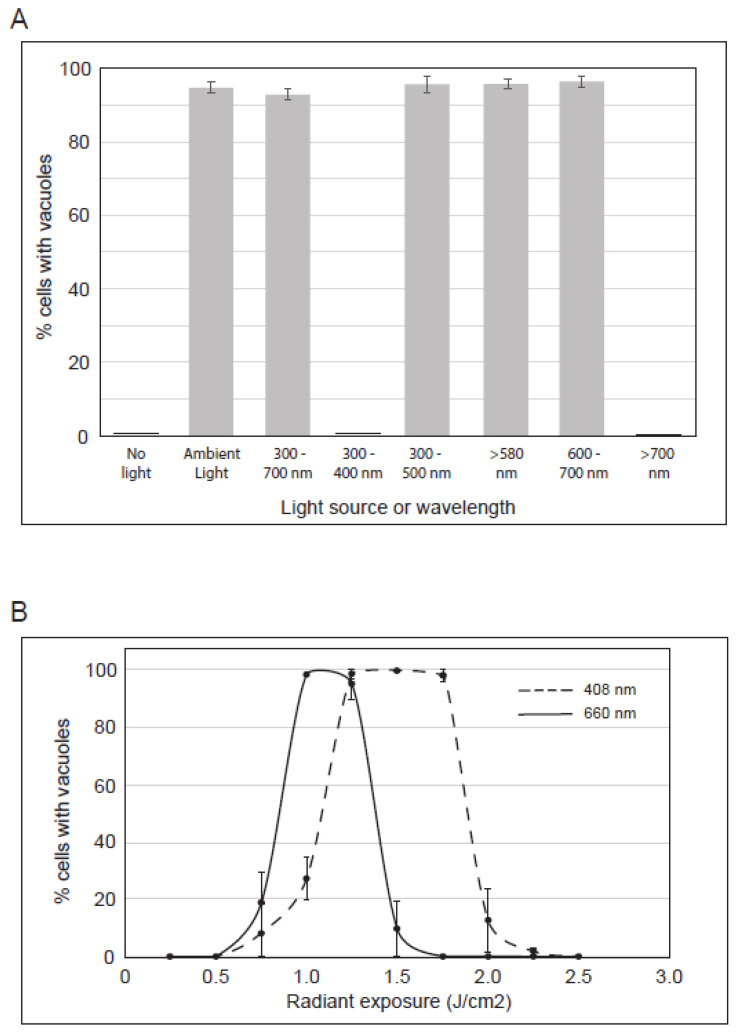
Characterization of the irradiation that induces vacuoles in cells treated with PP-630F. (**A**) U2OS cells were treated with PP-630F and not exposed to ambient light, exposed to ambient light, or exposed to ambient light filtered through optical glass of known transmission values. The light source and wavelengths (nm) are listed. The mean percentage of cells with vacuoles was calculated from three experiments and standard errors of the means are shown. (**B**) U2OS cells were treated with 25 µg/mL PP-630F and irradiated at increasing radiant exposures with LEDs at 408 nm (- - -) or 660 nm (—). The mean percentage of cells with vacuoles after each treatment was calculated at 16 h and standard errors of the means are shown.

We sought to better characterize the irradiation that induces vacuoles in PP-630F-treated cells. We used optical filters to limit the light spectrum during exposure (Figure 5A). Cells were either not treated or treated with PP-630F with no light, or cells were treated with PP-630F and ambient light that was filtered to permit the passage of distinct wavelengths. After 16 h cells were observed by phase-contrast microscopy and the percent of cells with vacuoles was determined. PP-630F-treated cells that were not exposed to light did not have vacuoles, whereas 96% of treated cells exposed to ambient light had vacuoles, as expected (Figure 5A). Similarly, 94% of cells had vacuoles when they were exposed only to the visible range of ambient light (400–700 nm), whereas those exposed to the ultraviolet range (300–400 nm) or the infra-red range (> 700 nm) of ambient light did not have vacuoles. Further sectioning of the visible spectrum with filters revealed that 97% of cells exposed to 300–500 nm wavelengths had vacuoles; 97% of cells exposed to 580 nm or higher wavelengths had vacuoles, and those exposed to wavelengths between 600–700 nm had 96% vacuolated cells. From these results we deduced that PP-630F treatment and concurrent exposure to light of 400–500 nm or 580–700 nm induce vacuolation in cells. 

We next tested energy levels and specific wavelengths using commercially available LEDs within the range we identified in Figure 5A. U2OS cells were not treated or treated with PP-630F and irradiated at increasing radiant exposures (J/cm^2^) with LEDs specific to either 408 nm or 660 nm (Figure 5B). In response to 1.25, 1.50, or 1.75 J/cm^2^ at 408 nm, PP-630F induced vacuolation in 98% of cells, whereas < 30% of cells showed a vacuolated phenotype at 0.75, 1, or 2 J/cm^2^, and no vacuolated cells were observed at 0.25, 0.5, 2.25, or 2.5 J/cm^2^. In response to 1 or 1.25 J/cm^2^ of 660 nm irradiation and PP-630F treatment, 95% of cells had vacuoles, whereas < 20% showed a vacuolated phenotype at 0.75 or 1.5 J/cm^2^, and no vacuolated cells were observed at 0.5 or lower, or 1.75 or higher J/cm^2^ (Figure 5B). These results revealed that vacuolation can be induced by PP-630F-treated cells with specific radiation and intensity.

**Figure 6:**
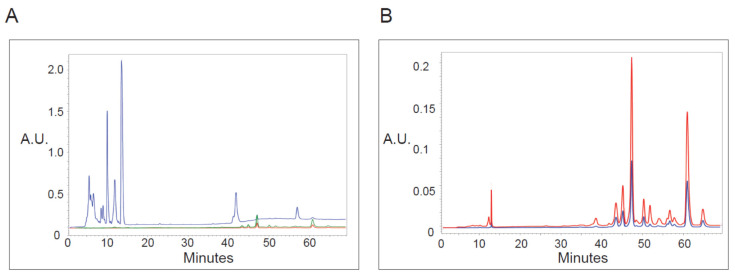
Analysis of PP-630 extracts by absorbance at various wavelengths. (**A**) PP-630F extracts were fractionated by HPLC C18 chromatography and absorbance (arbitrary units, A.U.) of eluted chemicals was monitored over time at wavelengths of 197 (blue), 410 (green), and 665 (red) nm. (**B**) PP-630F extracts were analyzed as described in A by higher absorbance sensitivity at 410 (green) and 665 (red) wavelengths. Note that the absorbance scale is different from that in A.

Knowing that irradiation at specific wavelengths was required to induce vacuoles, we asked if we could detect 410 or 665 nm absorbance within PP-630F extracts (Figure 6). Extracts were chromatographed by HPLC and absorbance was read at 197, 410, and 665 nm to detect UV absorbing chemicals. The extracts contained relatively strongly absorbing chemicals under 197 nm eluted between 5–15 min, with smaller peaks between 40 and 65 min (Figure 6A). By contrast, few peaks absorbing at 410 or 665 nm were detected before 45 min elution. However, minor peaks were detected by 410 and 665 nm absorbance eluted between 45 and 65 min, and those did not co-elute with UV absorbing peaks (Figure 6B). These data indicate that chemicals that do not absorb well at UV wavelengths but that absorb at 410 or 665 nm are present in the extract, which correlated with the irradiation wavelengths required for vacuole formation.

**Figure 7:**
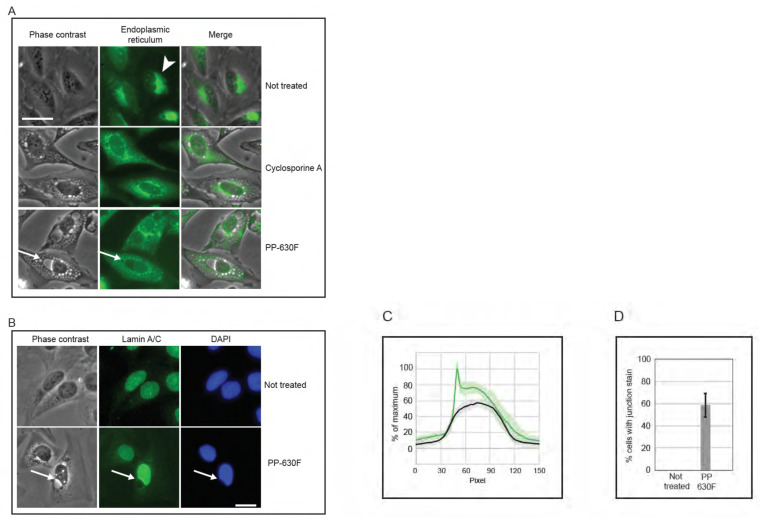
PP-630F and light-induced vacuolated cells show altered nuclear structures. (**A**) U2OS cells were not treated, treated with 20 µM cyclosporine A, or treated with 25 µg/mL PP-630F and exposed to ambient light. Immediately before imaging, cells were treated with endoplasmic reticulum stain Cytopainter. Cell images were collected by phase contrast microscopy (left panels) and by fluorescence microscopy (centre panels) and merged (right panels). Arrowheads indicate the endoplasmic reticulum in not-treated cells and arrows indicate a perinuclear vacuole in PP-630F-treated cells. Scale bar = 25 µm. (**B**) U2OS cells were either not treated or treated with 25 µg/mL of PP-630F and exposed to ambient light. Cells were imaged at 16 h by phase contrast and immunofluorescence microscopy using anti-lamin A/C antibodies and DAPI to mark nuclei. Arrows point to the vacuole–nucleus interface. Scale bar = 20 µm. (**C**) U2OS cells were treated as described in (B) and fluorescence signal intensity of lamin A/C was measured across nuclei in not-treated samples (black traces) or PP-630F-treated cells (green traces), which showed an intense staining at the junction of the nucleus and vacuole. Each treatment was run in triplicate and the mean and standard deviation of 45 nuclei are shown. Measurements were normalized to cytoplasmic intensity and the highest intensity across all treatments. (**D**) U2OS cells were treated as described in (B) and the mean percentage of cells with intense lamin staining at the perinuclear vacuole was calculated. The standard errors of the means from three experiments are shown.

We then compared vacuoles induced by PP-630F treatment to those induced by the natural product cyclosporine A (CsA), which had been reported to induce an endoplasmic reticulum-dependent, vacuolated phenotype [13,14]. Cells were not treated, treated with CsA, or treated with PP-630F and light. The endoplasmic reticulum was detected by staining with ER Cytopainter and vacuoles were observed by phase contrast and fluorescence microscopy (Figure 7A). Not-treated cells did not have vacuoles, whereas CsA-treated cells accumulated relatively small vesicles and a diffuse staining for the endoplasmic reticulum. At no time did CsA-treated cells acquire a large perinuclear vacuole. By contrast, perinuclear vacuoles were observed in PP-630F and light-treated cells. Strikingly, the endoplasmic reticulum staining was diffuse in these cells, in contrast to that of not-treated cells. These results revealed that the vacuolated phenotype for PP-630F and light-treated cells was different from that of CsA induced vacuoles.

We next investigated the position of the vacuole relative to the nucleus in cells treated with PP-630F and light (Figure 7B). U2OS cells were either not treated or treated with PP-630F and light, and they were examined by phase contrast microscopy and lamin A/C staining by immunofluorescence microscopy. Not-treated cells did not have a perinuclear vacuole and showed a uniform lamin A/C staining at the nucleus. By contrast, PP-630F and light-treated cells showed a prominent perinuclear vacuole that altered the lamin A/C staining. Treated cells had an indentation at the site where the vacuole joined the nucleus and the lamin A/C staining did not extend around the vacuole. Furthermore, treated cells appeared to have a higher intensity of lamin A/C staining across the nucleus. We were able to measure this intensity difference between not-treated and treated cells (Figure 7C) and found that 59% of treated cells showed a higher intensity of lamin A/C staining across the nucleus (Figure 7D). Strikingly, this staining was particularly intense at the nuclear–vacuole junction. These results suggest that the vacuoles induced by PP-630F and light target the cell immediately outside of the lamin component of the nucleus and create a change in morphology of the nucleus.

## 3. Discussion

This is the first report about the biological activity of extracts prepared from *Symphoricarpos occidentalis*, a plant found in the prairie ecological zone of Canada. *S. occidentalis* is a species of the small *Symphoricarpos* genus in the Caprifoliaceae (Honeysuckle) family. It is native to much of North America and is prevalent east of the Rocky Mountains in bush areas of the Alberta prairies, valley slopes, and coulees [10,15]. *Symphoricarpos* species show resistance to browsing by livestock due to their low palatability compared to other shrubs [16]. It is known to First Nations communities in Canada as a medicinal plant [17], and it is listed as a toxic plant in the Canadian Poisonous Plants Information System [18].

We used phenotypic assays to characterize the biological activity of *S. occidentalis* extracts. We observed that cells treated with extract PP-630F acquired an unusual vacuolated phenotype comprised of a prominent, large vacuole adjacent to the nucleus and smaller vesicles throughout the cytoplasm. The induction of the vacuole was dependent upon extract concentration and irradiation energy. Through the use of optical filters, we were able to identify 408 nm and 660 nm wavelengths at 1.25 J/cm^2^ energy levels that induced vacuoles in nearly 100% of treated cells. Interestingly, cells died when treated with PP-630F and irradiated at intensities higher than 1.5 J/cm^2^ for 408 nm or 1.75 J/cm^2^ for 660 nm wavelengths. A critical observation was that the nuclear vacuole phenotype was observed only when light and extract were applied in the presence of cells. In other words, if the extract was first irradiated and then applied to cells, no nuclear vacuoles were formed. We interpret this observation to suggest that a cellular target is modified by a photodynamic chemical in *S. occidentalis* extracts.

Photodynamic chemicals from plants have been previously identified. One of the best studied is hypericin from the species *Hypericum perforatum* (St. John’s wort; Hypericaceae). Hypericin is believed to target the endoplasmic reticulum and has not been reported to induce nuclear vacuoles [19,20]. We were also able to distinguish autophagy-type vesicles from the phenotype induced by *S. occidentalis* extracts by comparing them to the autophagy-inducing agent, CsA, which does not require irradiation to induce autophagy [14]. Notably, another plant in the family Caprifoliaceae, *Lonicera japonica* (Japanese honeysuckle), has been reported to induce phototoxicity [21]. The authors did not isolate the photoactive compound or report a vacuolated phenotype. We have characterized several other extracts from prairie plants in our laboratory such as PP-120 reported here (*Thermopsis rhombifolia*), PP-006 (*Gaillardia aristata*), and PP-360 (*Hymenoxys richardsonii*) and did not observe perinuclear vacuoles in cells treated under similar conditions (not shown).

To better understand the phenotype induced by *S. occidentalis* extract and light we examined lamin A/C staining in treated cells. Lamin A/C are intermediate filament proteins located between the chromatin and the membranes comprising the nuclear envelope. The retention of the lamin A/C staining within the nucleus suggests that PP-630-treated cells are not undergoing a type of cell death named nuclearphagy [22]. During nuclearphagy, the lamina extends into the adjacent vacuole resulting in a bud-light structure with nuclear lamina staining. By contrast, we observed an indentation at the junction of the nuclear vacuole and the nucleus.

This investigation is the first on nuclear vacuole-inducing activity from a plant source. The photodamage might be directed to proteins involved in maintaining the shape of the nuclear envelope. It is noteworthy that treated cells develop such a striking morphology upon treatment with PP-630F and light, rather than a general toxicity from a highly reactive chemical. This feature suggests a specific or small number of cellular targets affected by the active ingredient in PP-630F. The production of perinuclear vacuoles or nucleus-associated vacuoles (NAVs) in human cells has been associated with several human diseases [23,24]. We intend to identify the photoactive compound(s) in *S. occidentalis* and investigate the exact mechanism underlying vacuolation of the endoplasmic reticulum (ER) and the nuclear envelope. Insight into this connection may benefit future studies of the nuclear envelope and contribute to our understanding of cell biology by using potent natural products.

## 4. Experiment

### 4.1. Plant Collection

Aerial plant parts of *Symphoricarpos occidentalis* were collected when flowering by sustainable practices with permits in southern Alberta, Canada at North 49° 40’ latitude and West 112° 51’ longitude, at an elevation of approximately 900 metres during 2017 and 2018. Plant taxonomy was confirmed to species [10] and verified by Prof. John Bain, director of the University of Lethbridge Herbarium. A voucher specimen was provided to the Herbarium as #Golsteyn 630. Following harvest, plants were dried at room temperature and stored in light-protected bags.

### 4.2. Preparation of Plant Extracts

Extracts were prepared from leaves by grinding dried material to a fine powder and suspending it in either 10 times (volume/weight) 75% (*v*/*v*) ethanol in water (PP-630A) or in 100% dichloromethane (PP-630B) and stirred overnight at room temperature. The suspension was filtered under vacuum (Whatman, No. 1, Fisher Scientific, Toronto, ON, Canada) and the soluble fraction was dried with a rotary evaporator. Extracts were weighed, given a code number, and stored in the dark at room temperature until use. For sequential extractions, the insoluble plant material following an extraction was dried in a dark environment overnight at room temperature. The sequence of extraction was 75% (*v*/*v*) ethanol in water (PP-630D), 100% n-hexane (PP-630E), and 100% dichloromethane (PP-630F). Extracts were dissolved in dimethyl sulfoxide (DMSO) (Sigma-Aldrich; D2438) to concentrations of 50 or 100 mg/mL and tested. 

### 4.3. Cell Culture

The human cell lines U2OS, MDA-MB-231, A549, and M059K were maintained in Dulbecco’s Modified Eagle Medium (DMEM)/F-12 (Gibco; 11320-082) supplemented with 10% (*v*/*v*) heat-inactivated fetal bovine serum (FBS) (Gibco Thermo Fisher Scientific, Toronto, ON, CA #12484028), 2 mM Modified Eagle Medium non-essential amino acids (MEM-NEAA) (Gibco; 11140050), and 15 mM (4-(2-hydroxyethyl)-1-piperazineethanesulfonic acid; HEPES), pH 7.4. The HT-29 cell line was maintained in RPMI 1640 medium (Gibco; 21870-092) supplemented with 10% (*v*/*v*) heat-inactivated FBS (Gibco; 12484028) and 1.6 mM GlutaMAX (Gibco; 35050-061). Cells were grown at 37 °C in 5% CO_2_ with a humidified atmosphere and the media were changed every two-three days. HT-29 cells were plated at 1.0 × 10^6^ cells/75 cm^2^ flask and cultured for 48 h prior to treatment, and the remaining cell lines were plated at 5.0 × 10^5^ cells/75 cm^2^ flask and cultured for 48 h prior to treatment. Nocodazole (200 µg/mL; Sigma; M1404-10MG) and cyclosporine A (10 mM; Cayman Chemical; 59865-13-3) were dissolved in dimethyl sulfoxide and stored at −20°C. DMSO was added as a solvent vehicle control in the not-treated samples. Standard conditions for extract treatment were 25 µg/mL PP-630F and 20 min of light at 1 h post-treatment, unless stated otherwise. 

### 4.4. Cell Viability Assay

The cytotoxicity of *S. occidentalis* extracts on HT-29 and U2OS cells were measured by the MTT (3-((4,5)-dimethylthiazol-2-yl)-2,5-diphenyl-tetrazolium bromide) assay (Sigma-Aldrich, M2128). HT-29 cells were plated at 5 × 10^5^ cells/96-well culture plate and cultured at 37 °C for 48 h prior to treatment. U2OS cells were plated at 2.5 × 10^5^ cells/96-well culture plate and cultured at 37 °C for 48 h prior to treatment. All treatments were performed in triplicate and experiments were performed three times. At 96 h, 20 µL of MTT solution and 5 mg/mL MTT in phosphate buffered saline (PBS) (137 mM NaCl, 3 mM KCl, 100 mM Na_2_HPO_4_, 18 mM KH_2_PO_4_) were added to the media in each well and the plates were incubated at 37 °C for 3.5 h. The media were then aspirated and 100 µL MTT solvent (4 mM HCl, 0.1% (*v*/*v*) IGEPAL (octylphenoxypolyethoxyethanol), in isopropanol) was added to each well. Plates were placed on a shaker for 30 min in the dark and absorbance was measured at 590 nm using an Epoch microplate spectrophotometer (BioTek) operated by Gen 5.0 software. Data were normalized to 0.1% DMSO and nonlinear regression analysis was performed using log(inhibitor) versus normalized response using a variable slope calculated from the data. Cytotoxicity was reported in IC50 concentrations; the concentration of the compound or extract that reduced the absorbance of MTT by 50% by comparison to 0.1% (*v*/*v*) DMSO-treated cells and the mean IC50 values were calculated from three experiments. 

### 4.5. Light Irradiation

For ambient light incubations, treated cells were incubated in the cell culture room at an approximately 2-m distance from 4 fluorescent light bulbs (Philips). For specific wavelength incubations, treated cells were incubated in the culture room by ambient light and appropriate optical filters were placed over culture dishes. Transmission spectra of Schott filters were plotted according to filter transmission data provided by Schott. To obtain the transmission spectrum of the Leitz-Wetzlar K580 optical filter (>580 nm), the filter absorbance was measured using an Ultrospec 2100 pro UV/Visible spectrophotometer (GE Healthcare, Fisher Scientific, Toronto, ON, Canada) and the percent transmission calculated using

A = 2 − log_10_(%T) (1)

where A is the absorbance measured by the spectrophotometer and %T is the percent transmission. Curves were prepared using the SCHOTT filter calculation tool in Microsoft Excel software [25].

LED incubations were performed using LED emitters specific to 408 nm (LED Engin LZ4-00UB00-00U8) and 660 nm (LED Engin LZ4-00R208) at a distance of 10 cm. Radiant exposure (J/cm^2^) was determined based on irradiation time (30 s) and radiant flux of the cell culture dish area (W/cm^2^). Radiant flux (W) was determined based on the typical normalized radiant flux over electrical current curves provided by LED Engin. Electrical current was measured using a power supply (Agilent E3615A) for the 408 nm (1000 mA) and the 660 nm (850 mA) LED at 15 V and 9 V, respectively. Radiant exposure was then regulated using Realterm (Slashdot Media, San Diego, CA, USA, v. 2.0.0.70) software changing the LED duty cycle by pulse width modulation using a Teensy microcontroller board (SparkFun; DEV-13305, Niwot, CO, USA) and a metal-insulator-semiconductor field-effect transistor (MOSFET) (ON Semiconductor, Phoenix, AZ, USA; MTP3055VL) voltage relay. 

### 4.6. Light Microscopy

HT-29 cells or other cell lines were seeded at 5.0 × 10^4^ or 2.5 × 10^4^ cells per mL, respectively. Multi-well plates were prepared and incubated at 37 °C for 48 h prior to treatment. Images were either captured with an Infinity 1 camera operated by Infinity Capture imaging software (Lumenera Corporation, Ottawa, ON, Canada) on an Olympus CKX41 inverted microscope or a Cytation 5 cell imaging multi-mode reader (Biotek, Winooski, VT, USA) operated by Gen 5 software (v 3.01). Images were processed using Adobe Photoshop (Mountain View, CA, USA, CC 2015.0.0) or Image J software (ImageJ; 1.50f; https://imagej.nih.gov/ij/index.html).

### 4.7. Fluorescence and Immunofluorescence Microscopy

For live-cell fluorescence microscopy, U2OS cells were plated into multi-well plates and incubated at 37 °C for 48 h prior to treatment. Cells were incubated with 1X ER Cytopainter green (Abcam, Cambridge, MA, USA; 139481) for 15 min at 37 °C. Nuclei were co-stained with 1 µg/mL Hoechst 33342 (Sigma-Aldrich, Oakville, ON, Canada; B2261) for 15 min at 37 °C when indicated. Cells were observed with a Cytation 5 (Biotek) microscope using either an Olympus UPlanFL N 20× objective with 0.45 numerical aperture or an Olympus UPlanFL N 40× objective with 0.60 numerical aperture. Images were captured using an Infinity 3 camera operated by Gen5 software (v 3.01) for the Cytation 5 microscope. Images were prepared using either Adobe Photoshop (CC 2015.0.0) software or Image J software (ImageJ; 1.50f) and experiments were performed three times.

For immunofluorescence microscopy, U2OS cells were plated into multi-well plates and incubated at 37 °C for 48 h prior to treatment. Treated cells were fixed at room temperature for 20 min in 3% (*v*/*v*) formaldehyde diluted in PBS. Fixation was quenched with 50 mM NH_4_Cl in PBS and cells were permeabilized for five minutes using 0.2% (*v*/*v*) Triton X-100 in PBS then incubated for 30 min with 3% (*w*/*v*) bovine serum albumin (BSA) in PBS-T (0.1% (*v*/*v*) Tween-20 diluted in PBS). Cells were then incubated with primary antibody anti-lamin A/C (Santa Cruz, Dallas, TX, USA; sc-6215; 1:150) diluted in 3% (*w*/*v*) BSA in PBS-T overnight at 4 °C. After washing with PBS-T, cells were incubated for 1 h at room temperature with Alexa Fluor 488-conjugated anti-goat secondary antibody (Life Technologies-Sigma-Aldrich, Oakville, ON, Canada; A11055; 1:150) diluted in 3% (*w*/*v*) BSA in PBS-T. Nuclei were then stained with 300 nM DAPI (4′,6-diamidino-2-phenylindole) in PBS-T for 15 min. After washing with PBS-T, cells were imaged in PBS-T using a Cytation 5 (Biotek) microscope operated by Gen5 software (v 3.01). Images were prepared using either Adobe Photoshop (CC 2015.0.0) software or Image J software (ImageJ; 1.50f).

Images of treated U2OS cells stained with antibodies against lamin A/C were used to determine lamin A/C fluorescence intensity. Straight lines of 150 pixels in length were drawn across nuclei of 30 cells for each treatment and the lamin A/C fluorescence plot profiles were determined by measuring the intensity of each pixel using Image J software (ImageJ; 1.50f). Pixel intensity measurements were then normalized to the lowest and highest pixel intensity across all treatments. Measurements were grouped into intense or not intense staining based on the presence or absence of a lamin A/C peak in the plot profile, respectively. Mean plot profiles were prepared by calculating mean intensities of each pixel and standard deviations of three experiments.

### 4.8. High Pressure Liquid Chromatography (HPLC)

The PP-630F extract (100 mg) was fractionated by C_18_ reversed-phase HPLC using an InertSustain, 5 μm, 25 × 1 cm column, with a gradient of 60:40 (*v*/*v*) acetonitrile/water to 98:2 (*v*/*v*) acetonitrile/water at a flow rate of 2 mL/min. Absorbances were measured at 197, 410, and 665 nm.

### 4.9. Statistical Analysis

Experiments were performed at least three times. Data and statistical analyses were performed using Prism 5 software (GraphPad, San Diego, CA, USA; 5.04) and data were plotted as means from three separate experiments ± standard errors of the means using Microsoft Excel 2010 software. Data and statistical analyses of fluorescence microscopy intensity data were plotted as mean plot profiles ± standard deviation to show the deviation of intensity measurements in each sub-population of the selected cells. One-way ANOVA with Tukey’s post hoc analyses were performed on manual cell counts to calculate statistically significant differences between group means. IC50 concentrations were calculated by log(inhibitor) versus normalized response analysis using a variable slope calculated from the data.

## Data Availability

Data are presented in this manuscript or available from Dr. Roy Golsteyn (roy.golsteyn@uleth.ca).

## Abbreviations

BSA	bovine serum albumin
CPT	camptothecin
CsA	cyclosporine A
DAPI	4′,6-diamidino-2-phenylindole
DMSO	dimethylsulfoxide
h	hours
LED	light emitting diode
min	minute
MTT	(3-((4,5)-dimethylthiazol-2-yl)-2,5-diphenyl-tetrazolium)
nm	nanometre
Noco	nocodazole
NT	not-treated
PBS	phosphate buffered saline
v	volume
